# Physicians using spinal manipulative treatment in The Netherlands: a description of their characteristics and their patients

**DOI:** 10.1186/s12891-017-1863-z

**Published:** 2017-12-06

**Authors:** Wouter Schuller, Raymond W. J. G. Ostelo, Daphne C. Rohrich, Adri T. Apeldoorn, Henrica C. W. de Vet

**Affiliations:** 1 VU University Medical Center, Department of Epidemiology & Biostatistics and the Amsterdam Public Health Research Institute, P.O. box 7057, 1007 MB Amsterdam, The Netherlands; 2 Spine Clinic, Mahoniehout 10-12, 1507 ED Zaandam, The Netherlands; 3 Department of Health Science of the Faculty of Earth and Life Sciences and the Amsterdam Public Health Research Institute, P.O. box 7057, 1007 MB Amsterdam, The Netherlands; 4 Rehabilitation department, Noordwest Ziekenhuisgroep, Wilhelminalaan 12, 1815 JD Alkmaar, The Netherlands

**Keywords:** Spinal manipulative treatment, Musculoskeletal medicine, MSK medicine, Patient characteristics, Chiropractic treatment, Manual therapy

## Abstract

**Background:**

Various health care professionals apply Spinal Manipulative Treatment (SMT) in daily practice. While the characteristics of chiropractors and manual therapists and the characteristics of their patient populations are well described, there is little research about physicians who use SMT techniques. A distinct group of physicians in The Netherlands has been trained in musculoskeletal (MSK) medicine, which includes the use of SMT. Our objective was to describe the characteristics of these physicians and their patient population.

**Methods:**

All registered MSK physicians were approached with questionnaires and telephone interviews to collect data about their characteristics. Data about patient characteristics were extracted from a web-based register. In this register physicians recorded basic patient data (age, gender, the type and duration of the main complaint, concomitant complaints and the type of referral) at the first consultation. Patients were invited to fill in web-based questionnaires to provide baseline data about previous treatments and the severity of their main complaint. Functional impairment was measured with Patient Reported Outcome Measures (PROMs).

**Results:**

Questionnaires were sent to 138 physicians of whom 90 responded (65%). Most physicians were trained in MSK medicine after a career in other medical specialities. They reported to combine their SMT treatment with a variety of diagnostic and treatment options part of which were only permissible for physicians, such as prescription medication and injections. The majority of patients presented with complaints of long duration (62.1% > 1 year), most frequently low back pain (48.1%) or neck pain (16.9%), with mean scores of 6.0 and 6.2, respectively, on a 0 to10 numerical rating scale (NRS) for pain intensity. Mean scores on all PROMs showed moderate impairment. Patients most frequently reported previous treatment by physical therapists (68.1%), manual therapists (37.7%) or chiropractors (17.0%).

**Conclusion:**

Our study showed that MSK physicians in The Netherlands used an array of SMT techniques. They embedded their SMT techniques in a broad array of other diagnostic and treatment options, part of which were limited to medical doctors. Most patients consulted MSK physicians with spinal pain of long duration with moderate functional impairment.

## Background

Spinal Manipulative Treatment (SMT) is used world-wide to treat musculoskeletal problems such as low back pain and neck pain [1]. Given the socioeconomic impact of these conditions and the wide spread use of SMT, determining the efficacy of SMT is a priority for all health care stakeholders. However, determining the efficacy of SMT is challenging. Cochrane reviews for SMT in the treatment of neck pain and chronic low back pain have concluded that there is evidence for some effect, but the size of this effect is small [2–4]. Outcomes may be influenced by the heterogeneity of the patient population, or by the clinical setting wherein SMT is used. That is, it is possible that SMT is only effective in subgroups of patients, or that the efficacy is influenced by the variety of clinical settings in which SMT techniques are applied by various health care professionals [5]. Whilst SMT is generally associated with chiropractors and manual therapists, SMT techniques are also applied by groups of specially trained physicians. Currently, characteristics of chiropractors and manual therapists and their patients are well described [6–17]; however, little is known about physicians trained in the use of SMT [18].

In The Netherlands, there is a group of physicians who have been trained in musculoskeletal (MSK) medicine, including the use of SMT. These physicians are titled “physician for musculoskeletal medicine” and united in the Dutch Association for Musculoskeletal Medicine. To obtain registration as a physician for MSK medicine a two year training program consisting of both theoretical and practical work must be successfully completed after qualifying as a medical doctor. The theoretical component covers specialist knowledge of manipulative treatments, orthopaedics, neurology, radiology, epidemiology, research methodology and medico-legal aspects. The practical training consists of working as a trainee at a designated training practice for at least two days a week for a period of two years. During this time the trainee specializes in at least one of two types of SMT techniques. One SMT technique, manual medicine, is mainly derived from chiropractic and manual therapeutic techniques, diagnosing and correcting limitations in segmental motion. The other SMT technique, orthomanual medicine, has been developed more recently in The Netherlands, and identifies and corrects alterations in joint positions. These joint positions are considered to be interconnected throughout the spine, and are corrected in a strict sequence of specific mobilizing techniques. The technique has been shown to differ from manual therapy and chiropractic treatment [18].

The objective of our study was to describe the characteristics of physicians for musculoskeletal medicine in The Netherlands and the characteristics of their patients.

## Methods

### Study design

We conducted a descriptive study of the characteristics of Dutch MSK physicians and their patients. All members of the Dutch Association for Musculoskeletal Medicine (*N* = 138) were invited to participate. First, we contacted the physicians by mail to participate in a survey to collect physician characteristics. In addition, we contacted all physicians by telephone to stimulate response. We asked participating physicians to provide written informed consent. Second, we asked physicians to participate in a web-based patient registry and to invite all consecutive patients who presented for the first time in MSK practice. If patients gave informed consent, the treating physician entered email addresses of the recruited patients in the registry. Thereafter, we used a specially designed computer program (Readmail) to automatically distribute invitations to patients by email to fill in web-based questionnaires.

During three consecutive time periods, this registry was used to collect data about patient characteristics. In each period different sets of outcome measures were used, resulting in three cohorts of patients with specific sets of outcome measures (Table 1).

**Table 1:** Overview of physician and patient related data collection

Type of data	Source of data	Outcome measures	
Physician characteristics	Survey	Demographics, training, treatment and referral patterns	
	Telephone call	Number of days per week spent in MSK practice	
Patient characteristics (web-based registry)	**Sample**	**Patient questionnaires**	**Treating physician**
	Cohort 1 (09/12–03/13)	Numerical Rating Scale	Demographics, source of referral, type and duration of complaints, and treatment
	Cohort 2 (04/13–01/14)	RDQ, NDI, LEFS, DASH, HIT-6^a^	
	Cohort 3 (02/14–02/16)	Previous treatments	

^a^RDQ (Roland Disability Questionnaire); 24 items, range 0–24, higher scores indicate more disability

*NDI* (Neck Disability Index); 10 items, range 0–50, higher scores indicate more disability

*LEFS* (Lower Extremity Function Scale); 20 items, range 0–80, higher scores indicate less disability

*DASH* (Disabilities of the Arm, Shoulder, and Hand); 30 items, range 0–100, higher scores indicate higher disability

*HIT-6* (Headache Impact Test); 6 items, range 36–78, higher scores indicate more disability

### Data collection of physician characteristics

We collected data about physician characteristics using a paper survey sent by mail. In this survey, physicians were asked about their age, gender, their medical background, additional training in other medical specialties, the use of specific techniques and cooperation with other healthcare providers. In addition, we contacted all physicians by telephone to collect data about the number of days per week spent in MSK practice (Table 1).

### Data collection of patient characteristics

Both the treating physician and the individual patients provided data, which were recorded in the web-based registry (Table 1). The treating physicians registered the following baseline data of patients: age, gender, type and duration of the main complaint, and the existence of concomitant complaints. The treating physicians coded the main and concomitant complaints according to the International Classification of Primary Care (ICPC) [19].

Three consecutive cohorts of patients were presented with three different sets of baseline and outcome measures. The first cohort of patients provided information about the pain intensity on a Numerical Rating Scale (NRS). The second cohort provided data regarding functional limitations due to their main complaint. Patients with low back pain completed the Roland-Morris Disability Questionnaire (RDQ), patients with neck pain completed the Neck Disability Index (NDI), patients with upper extremity complaints completed the Disabilities of the Arm, Shoulder and Hand (DASH) questionnaire, patients with lower extremity complaints completed the Lower Extremity Function Scale (LEFS), and patients with headache or migraine completed the Headache Impact Test (HIT-6). All instruments are commonly used in research and have been validated in Dutch populations [20–26]. The third cohort provided data about previous treatments.

### Data analyses

We analyzed data using descriptive statistics in SPSS, version 22.

## Results

### Characteristics of physicians

Our survey was sent to all 138 members of the Dutch Association for Musculoskeletal Medicine, and returned by 90 physicians (65%). One physician did not tick the informed consent box and was removed from the analyses. Physician characteristics are presented in Table 2. After finishing medical training and before training in MSK medicine the majority of MSK physicians had worked in other medical specialties. Some had finished specialist training in other fields, most frequently in general practice (32.2%) or occupational medicine (16.7%). Of the two SMT techniques taught in the training program, a higher proportion of physicians had finished training in the manual medicine technique (63.3%) than the orthomanual medicine technique (58.9%). A number of MSK physicians were familiar with other musculoskeletal treatment options, for example, McKenzie [27–30] or the use of protocols developed by the Spine Intervention Society (SIS) [30–33].

**Table 2:** Physician characteristics

Number of registered MSK physicians	138
Number of respondent	90 (65%)
**Demographics**	
Gender (male)	77.5%
Age (range)	57 (38–75)
MSK consultations per week (range)	51 (5–150)
> 3 days in MSK practice	57.3%
**Background training (%)**	
Trained as General Practitioner	32.2
Still registered as General Practitioner	12.2
Trained in occupational medicine	16.7
Still registered in occupational medicine	8.9
**MSK training (finished training, %)**	
Orthomanual technique	58.9
Manual technique	63.3
McKenzie	13.3
Marsman	13.3
Spine Intervention Society	11.3

Table 3 presents an overview of treatments used by MSK physicians, as reported by the physicians in our survey. SMT techniques were used predominantly. Although a higher proportion of physicians had followed training in the manual technique, the orthomanual technique was used more frequently in daily practice (used often or regularly in 70.6% versus 56.2%). Regular use of McKenzie treatment was reported by 41.7% of respondents. Other commonly used supportive treatment options were training advice (e.g. advice on sports activities that could support the treatment) and postural advice (e.g. advice about how to perform ADL activities). Regular use of general medical injections (e.g. steroid injections for acute bursitis of the shoulder), prescription medication, and injection treatment according to SIS guidelines under X-ray guidance was reported by 34.8%, 37.1%, and 15.3% of respondents, respectively. Complementary treatment such as homeopathy or acupuncture was used regularly by less than 8% of the respondents.

**Table 3:** Self-reported treatments used in daily practice by 89 Musculoskeletal physicians

Technique	Never/Seldom (%)	Sometimes (%)	Regular/Often (%)
**Spinal Manipulative Treatment**			
Orthomanual medicine technique	20.0	9.4	70.6
Manual medicine technique	20.2	23.6	56.2
McKenzie	29.8	28.6	41.7
Marsman	66.3	17.5	16.3
**Supportive Treatments**			
Training advice	9.0	15.7	75.3
Postural advice	4.4	12.2	83.3
Dietary advice	42.5	33.3	24.1
Prescribed medication	25.8	37.1	37.1
**Injections**			
Injections general medical	43.8	21.3	34.8
Injections SIS	82.4	2.4	15.3
Injections trigger point	70.1	16.1	13.8
Injections neural therapy	69.0	20.7	10.3
**Complementary Treatments**			
Homeopathy	84.1	11.4	4.5
Acupuncture	87.4	5.7	6.9
Dry needling	87.2	5.8	7.0
Podology	81.4	10.5	8.1

Referral patterns, reported by the physicians in the survey, are presented in Table 4. Regular referral to physical therapy, exercise therapy, and postural therapy was reported by 46.1%, 62.2%, and 47.1% of the responding physicians, respectively. Physicians also reported further referral to other MSK physicians (referral from manual medicine to orthomanual medicine 20.5%, referral from orthomanual medicine to manual medicine 16.7%). Regular cooperation with medical specialists was mainly reported for orthopaedics, neurology and (anaesthetic) pain clinics (30.3%, 25.6%, and 28.7% respectively).

**Table 4:** Referral of MSK physicians (*N* = 89) to other specialists and practitioners

Specialism	Never/Seldom (%)	Sometimes(%)	Regular/Often (%)
**SMT**			
Orthomanual medicine technique	47.0	32.5	20.5
Manual medicine technique	66.7	16.7	16.7
Chiropractor	96.4	2.4	1.2
Manual therapist	66.7	27.2	6.2
McKenzie	47.0	28.9	24.1
Marsman	84.1	13.4	2.4
**Supportive treatment**			
Physiotherapy	16.9	37.1	46.1
Exercise therapy	5.7	32.2	62.1
Postural therapy	12.6	40.2	47.1
Dietician	61.9	31.0	7.1
**Medical specialists**			
Neurologist	12.2	62.2	25.6
Orthopaedic surgeon	16.9	52.8	30.3
Rehabilitation	64.7	25.9	9.4
Pain clinic/ SIS	36.8	34.5	28.7
**Complementary treatments**			
Trigger point therapy	90.4	8.4	1.2
Neural therapy	91.6	7.2	1.2
Homeopathy	82.1	16.7	1.2
Acupuncture	79.5	18.1	2.4
Dry needling	83.3	15.5	1.2
Insoles	41.4	37.9	20.7

### Characteristics of patients

A group of 31 MSK physicians volunteered to register patient data in our web-based registry, and to recruit patients. Demographic characteristics of the participating physicians (79% male, average age 54) were comparable to the demographic characteristics of both the whole population of MSK physicians (81% male, average age 57) and the part of the population that had answered to the physician survey (79% male, average age 56). Patient characteristics are presented in Table 5. The first cohort consisted of 1704 patients, of whom 1498 completed a baseline questionnaire (80%). The data registered by the treating MSK physician showed that 42 % of patients were male, and the predominant main complaint was low back pain without sciatica (30.0%), followed by low back pain with sciatica (18.1%) and neck pain (16.9%). Most patients (62.1%) had a main complaint that had been present for more than one year, only 16.3% had a main complaint that had lasted for less than three months. More than half of the patients (61.0%) sought care through self-referral, while 16% was referred by a general practitioner. The baseline questionnaire answered by the patients showed average NRS scores for the subgroups of patients with low back pain, neck pain and other complaints of 6.0, 6.2, and 6.0, respectively.

**Table 5:** Patient characteristics

**Cohort 1 data**	**N**	**Main Complaint (ICPC code)**	**Percent**
Number of registrations	1704	Spine (L2, L3, and L86)	73.9
Number of respondents	1498	Low Back without sciatica (L3)	30.0
		Low Back with sciatica (L86)	18.1
		Neck (L1)	16.9
		Headache (N01, N02, and N89)	4.6
		Upper Extremity (L8-L12)	7.3
		Lower Extremity (L13-L17)	8.6
		Other	5.6
		**Duration**	
		< 3 months	16.3
		3–12 months	21.6
		> 1 year	62.1
		**Source of referral**	
		General practitioner	16.1
		Physiotherapist	8.7
		Medical Specialist	3.2
		Self-referral	61.0
		Other	11.0
		**NRS pain** ^**a**^	**Mean (sd)**
		Low Back (*N* = 722)	6.0 (2.0)
		Low Back without sciatica (*N* = 449)	5.9 (1.9)
		Low Back with sciatica (*N* = 273)	6.2 (2.0)
		Neck (*N* = 250)	6.2 (2.0)
		Other (*N* = 526)	6.0 (2.2)
**Cohort 2 data**		**Function measures** ^**a**^	**Mean (sd)**
Number of registrations	2610	RDQ (*N* = 827)	8.9 (5.3)
Number of respondents	1701	NDI (*N* = 269)	13.1 (7.2)
		LEFS (*N* = 159)	55.0 (15.8)
		DASH (*N* = 102)	31.6 (16.5)
		HIT-6 (*N* = 54)	60.0 (7.5)
**Cohort 3 data**		**Previous treatments**	**Percent**
Sample of respondents	433	Physical therapy	68.1
		Manual therapy	37.7
		Chiropractic treatment	17.0
		MT or chiropractor	45.9
		MT and chiropractor	8.8
		Medication	25.6
		Injections (pain clinic)	6.7
		Surgery	4.4

^a^Patient Reported Outcome Measures were tailored to the main complaint

The second cohort consisted of 2610 patients, of whom 1701 patients answered to a baseline questionnaire (65%). Average baseline scores on the specific functional PROMs showed a moderate level of functional disability.

A sample of 433 patients was extracted from the third cohort, in which patients provided data about previous treatments. The majority of patients (82.1%) had been treated otherwise before consulting a MSK physician. Patients most frequently reported previous treatment by physical therapists (68.1%), followed by manual therapists (37.7%), medication (25.6%) and chiropractors (17.0%). Almost half (45.9%) of the patients had previously been treated by manual therapists or chiropractors, and 8.8% had been treated by both manual therapists and chiropractors.

## Discussion

While the characteristics of chiropractors and manual therapists and their patients are well described, little is known about MSK physicians who use SMT. Our study is a first step to address this knowledge gap: we described MSK physicians in The Netherlands and their patients. Most MSK physicians in The Netherlands had previous experience in other medical specialties. They were trained in a variety of SMT techniques that, in part, differed from the techniques used by chiropractors and manual therapists. Furthermore, they used an array of other diagnostic and treatment options, part of which were, by law, restricted to medical doctors, such as prescription medication and general medical injections or injections under X-ray guidance. Physicians reported frequent use of training advise or postural advise and further referral for exercise therapy or physiotherapy.

The majority of patients consulting MSK physicians reported spinal pain of long duration, with moderate functional disability. This is comparable to the patient population reported in a previous study to consult chiropractors in The Netherlands [17]. Patients consulting manual therapists [16] reported, on average, musculoskeletal pain of shorter duration (59% < 3 months, 21% > 1 year) than the patients seen by chiropractors (24% < 3 months, 58% > 1 year) and MSK physicians (16% < 3 months, 62% > 1 year). Patients consulting MSK physicians had frequently been treated previously by other SMT professionals. This could be reflective of the practice in The Netherlands where, traditionally, the general practitioner refers patients with musculoskeletal complaints to physical therapists. In The Netherlands, manual therapy is a subspecialty of physical therapy, and thus manual therapists are likely to be consulted by patients with less severe complaints at an earlier stage. Only when complaints are refractory to treatment do patients consult chiropractors or MSK physicians. This practice is supported by health care insurance policies, which generally cover a number of physiotherapy treatments; the costs of chiropractic care and MSK medicine are only reimbursed for patients with additional coverage.

It must be noted that our study described the situation in The Netherlands. Due to differences in health care organization, recognition of the various professional groups and reimbursement of the costs of treatment, respective patient populations may vary between countries. In Denmark, for example, chiropractic treatment is embedded in regular primary care, with strong academic connections [34], while in The Netherlands and Belgium chiropractic treatment is considered to be complementary medicine [13, 17]. Furthermore, in other countries, the various professional groups might have different licensing requirements for prescribing medication or applying injections. Comparable variations exist in the position of MSK physicians. MSK medicine is practised in other European countries as an additional competence to other medical specialities, while in The Netherlands it is put forward as a medical profession in its own right.

### Strengths and limitations

The main strengths of this study are that the whole population of physicians registered in MSK medicine was approached for our study, and the large number of patients who provided data. Nearly all physicians using SMT in The Netherlands are members of the Dutch Association for Musculoskeletal Medicine, because registration in the Register for Musculoskeletal Medicine is necessary to have the costs of treatment reimbursed, and this registration can only be obtained after completing the professional training program. A limitation of our study could be that only 65% of the members returned our survey. However, demographic characteristics (age and sex) of the responding physicians were comparable to non-responders. Another limitation could be that data on physician characteristics was self-reported. Lastly, we obtained patient data from a subset of MSK practices, as not all MSK practices were willing to collect patient data. However, we consider the data to be representative as the demographic characteristics of the participating physicians were comparable to the demographic characteristics of all members of the Dutch Association for Musculoskeletal Medicine.

### Further study

Additional studies describing physicians who are trained to use SMT in other countries are needed. There are differences in the type of SMT technique used by various professionals [18]. Future studies should clearly report the SMT techniques in detail. The CIRCLe SMT study presented criteria for reporting SMT techniques [35]. Lastly, studies in which various SMT techniques are embedded within different treatment protocols are warranted.

## Conclusion

MSK physicians in The Netherlands reported to use an array of SMT techniques. They had embedded their SMT techniques in a broad array of other diagnostic and treatment options, part of which were limited to medical doctors. Most patients consult MSK physicians with spinal pain of long duration with moderate functional impairment.

## Abbreviations

CIRCLe SMT: Consensus on interventions reporting criteria list spinal manipulative therapy
DASH: Disabilities of the arm, shoulder and hand
HIT-6: Headache Impact Test
ICPC: International classification of primary care
LEFS: Lower extremity function Scale
MSK: Musculoskeletal
NDI: Neck disability index
NRS: Numerical rating scale
RDQ: Roland morris disability questionnaire
SIS: Spine intervention society
SMT: Spinal manipulative treatment
